# Muscle MRI in a Rare Case of Limb-Girdle Muscular Dystrophy 1B

**DOI:** 10.7759/cureus.72413

**Published:** 2024-10-26

**Authors:** Siddharth S Bokil, Eshan Chetan Durgi, Rohan N Shah, Amanya Shukla, Prashant Rawat

**Affiliations:** 1 Radiodiagnosis, Dr. D. Y. Patil Medical College, Hospital & Research Centre, Dr. D. Y. Patil Vidyapeeth (Deemed to be University), Pune, IND

**Keywords:** limb-girdle muscle dystrophy, mri, muscle atrophy, muscle weakness, thigh muscles

## Abstract

The term limb-girdle muscular dystrophy (LGMD) refers to a variety of genetic neuromuscular disorders that typically affect the proximal muscles surrounding the hip and shoulder girdles. Despite having multiple genetic subtypes, these share similar clinical and imaging findings. Autosomal dominant forms are grouped under type 1, and autosomal recessive forms are grouped under type 2. Limb-girdle muscle dystrophy 1B (LGMD1B) is an autosomal dominant form. It has a variable age of onset. It is caused by a mutation in the Lamin A/C gene. A 60-year-old male presented with a history of slowly progressive bilateral lower limb weakness. Laboratory tests revealed elevated levels of serum creatinine kinase. He underwent a magnetic resonance imaging (MRI) of bilateral hips and thigh regions. MRI revealed moderate to severe fatty infiltration of the muscles of the hip and thigh regions in a bilaterally symmetrical fashion. Further testing confirmed the diagnosis of LGMD1B.

## Introduction

The term limb-girdle muscular dystrophy (LGMD) refers to a variety of genetic neuromuscular disorders that typically affect the proximal muscles surrounding the hip and shoulder girdles [1]. They usually show characteristic involvement of the proximal group of muscles, with a highly variable age of onset [2]. MRI, in tandem with clinical evaluation, may play a role in selecting appropriate genetic tests and more generally in the differential diagnosis of genetically distinct forms of neuromuscular disorders. Limb-girdle muscle dystrophy 1B (LGMD1B) is an autosomal dominant form, which often involves the wasting of hip, shoulder, and thigh muscles. Although there are no specific treatments currently available for LGMD1B, proper diagnosis and symptomatic management play a major role in the improvement of quality of life. Through this case, we aim to highlight the importance of recognizing the pattern of muscle involvement on imaging in LGMD, which has the potential to facilitate earlier diagnosis of the disease; to highlight the role of MRI in the evaluation of the degree of muscle involvement, which plays a major role in the assessment of the physical limitations due to muscle wasting; and to plan the physical rehabilitation of the patient accordingly. Early diagnosis is crucial, as it leads to further evaluation of the patient for associated life-threatening abnormalities.

## Case presentation

A 60-year-old male patient presented to our hospital with complaints of slowly progressive weakness of the bilateral lower limbs in the last eight years. Currently, the patient has difficulty walking and needs support to walk. The patient was not aware of similar complaints in his family. Laboratory tests revealed a mildly elevated serum creatinine kinase. A two-dimensional (2D) echo revealed mild features of dilated cardiomyopathy; however, an electrocardiogram (ECG) did not reveal any evidence of conduction abnormalities. Subsequently, the patient underwent an MRI of the hip and bilateral thighs.

**Figure 1:**
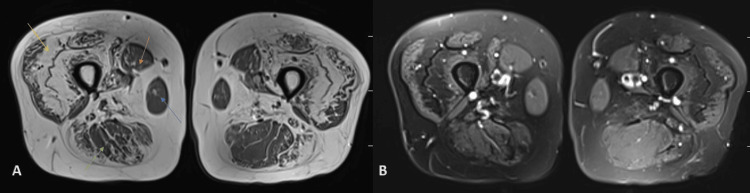
MRI of the bilateral thigh region showing fatty infiltration of muscles. Figure 1A: T1-weighted MRI of the bilateral thigh region shows fatty infiltration (exhibiting a high T1 signal) in a bilaterally symmetric fashion, involving the anterior group of muscles (yellow arrow) and posterior group of muscles (green arrow). Sartorius (orange arrow) and gracilis (blue arrow) are spared. Figure 1B: T2-weighted fat-saturated image at the corresponding level showing suppression of the fat-related signal. Also, no evidence of edema was noted.

**Figure 2:**
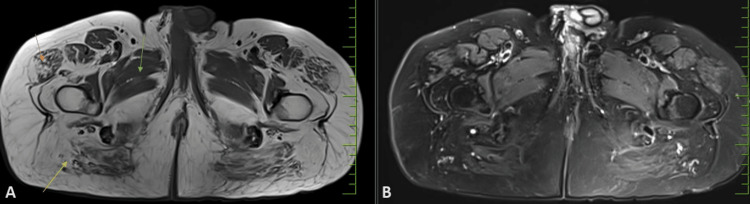
MRI of the bilateral lower hip region showing fatty infiltration of muscles. Figure 1A: T1-weighted MRI showing fatty infiltration in a bilaterally symmetric fashion, involving the gluteus muscles (yellow arrow), tensor fascia lata (orange arrow), and relative sparing of the adductor group of muscles (green arrow). Figure 1B: T2-weighted fat saturated image at the corresponding level showing suppression of fat signal. No changes in edema seen.

MRI showed evidence of moderate to severe fatty infiltration (exhibiting a high T1/T2 signal) of the muscles of the hip and thigh regions in a bilaterally symmetrical fashion (Figures 1, 2). The affected muscle groups include the gluteus muscles including the maximus, medius, and minimus muscles (Figure 2); anterior muscle groups that include the vasti, rectus femoris, and tensor fascia lata (Figure 1); and posterior muscle groups that include the semimembranosus, semitendinosus, and biceps femoris (Figure 1). It was seen that there is a relative sparing of the adductor group of muscles, sartorius and gracilis muscles (Figure 2); however, these showed changes in atrophy. The obturator group of muscles including the obturator internus and extrernus and pectineus, quadrates femoris, and ilio-psoas were unremarkable. Visualized bones showed a normal signal intensity. There was no evidence of muscle edema.

Since there are no specific treatments for LGMD1B, the patient was referred to the Department of Orthopedics for symptomatic management, which included regular physiotherapy. The patient was also referred to the Department of Cardiology for the management of dilated cardiomyopathy and subsequent follow-up. The patient is currently undergoing physiotherapy and is under follow-up with the Department of Cardiology.

## Discussion

LGMDs are characterized by muscle weakness that preferentially involves proximal muscle groups. The age of onset and disease severity are highly variable. Autosomal recessive forms are generally more common and usually present during childhood. By contrast, autosomal dominant forms typically have an adult onset but can also manifest during the teenage years. Autosomal dominant LGMDs are designated as type 1, and autosomal recessive LMGDs are designated as type 2. LGMD1B is an autosomal dominant form of LGMD. It is caused by a mutation in the Lamin A/C gene, which is involved in muscle fiber protein production [3]. From an imaging perspective, initial evaluation with ultrasound may allow us to evaluate fatty infiltration of muscles based on their echogenicity; however, MRI is the modality of choice as it has superior soft tissue contrast, which can help evaluate atrophy and pseudohypertrophy [4]. T1-weighted images are preferred for the evaluation of relative fatty infiltration. STIR/T2-weighted images help detect the presence of edema within the muscles. Wen-Chi Hsu et al. [5] conducted a study to recognize muscle MRI patterns to differentiate LGMD from other clinically similar conditions like idiopathic inflammatory myopathy (IIM). They found a significantly higher grade of fat substitution in the muscles of the thigh. In comparison, fewer patients showed high-grade fat substitution in IIM. They also reported that patients with IIM had more severe muscle edema in comparison to LGMD, especially in the adductor magnus and soleus.

Díaz-Manera et al. [6] conducted a review study on the role of muscle MRI in muscular dystrophies. They reported on the pattern of muscles involved in particular types of LGMD. In their review study, regarding LGMD1B, they reported that the gluteus minimus and gluteus medius appeared to be involved in the majority of patients. This was in concurrence with the present case. Among the thigh muscles, they reported predominant involvement of the posterior muscles of the thigh, especially semimembranosus and adductor muscles. In the present case, semimembranosus was involved, but the adductor group of muscles was relatively spared. They also reported that the sartorius and gracilis were normally not involved, which was in concurrence with the present case.

Patients with LGMD1B have a risk of associated cardiac problems, predominantly dilated cardiomyopathy and dysrhythmia [7]. It is important to assess the cardiac function in these patients as this can become a major factor affecting life expectancy.

The treatment of LGMD1B is predominantly symptomatic as no specific treatment is currently available [3]. Regular physiotherapy and adequate cardiology follow-ups are recommended. Detection of specific patterns on MRI may aid in the early diagnosis of cardiac problems, potentially increasing life expectancy in these patients.

The literature on evaluating patterns of muscle involvement in various types of LGMDs is scarce, especially for the rare types like LGMD1B. Further research is required to better understand and differentiate various types on imaging alone.

## Conclusions

LGMD1B is an autosomal dominant form of LGMD. A diagnosis of muscular dystrophy depends on various parameters like clinical, pathological, and biochemical parameters. Muscle MRI is increasingly becoming an essential tool for evaluating muscular dystrophy. Our case highlights the importance of recognizing the pattern of muscle involvement. Early diagnosis is crucial to further evaluate the associated conditions, commonly involving the cardia, which can have a significant impact on life expectancy. A definitive diagnosis is possible only after genetic testing. This case aims to highlight the importance of recognizing certain patterns of muscle involvement, which can aid in the diagnosis of LGMD.
